# Comparing Clearances of Polymethylmethacrylate and Polysulfone Dialyzer Membranes for Postdilution Hemodiafiltration in Patients With Kidney Failure: Protocol for a Systematic Review and Meta-Analysis

**DOI:** 10.2196/71943

**Published:** 2025-06-26

**Authors:** Cahyani Gita Ambarsari, Astrid Kristina Kardani, Agustina Wulandari, Nadhifah Nadhifah

**Affiliations:** 1 Department of Child Health Faculty of Medicine Universitas Indonesia Cipto Mangunkusumo Hospital Central Jakarta Indonesia; 2 School of Medicine University of Nottingham Nottingham United Kingdom; 3 Medical Technology Cluster Indonesian Medical Education and Research Institute (IMERI), Faculty of Medicine Universitas Indonesia Jakarta Indonesia; 4 Nephrology Division, Department of Child Health Dr Saiful Anwar General Hospital, Faculty of Medicine Universitas Brawijaya, Malang East Java Indonesia; 5 Division of Nephrology, Department of Child Health Faculty of Medicine Universitas Sebelas Maret Surakarta Indonesia; 6 Division of Nephrology Department of Child Health Dr Moewardi Hospital Surakarta Indonesia; 7 Faculty of Medicine Universitas Airlangga Surabaya Indonesia

**Keywords:** albumin, beta 2-microglobulin, creatinine, dialysis, end stage kidney disease, polymethyl methacrylate, polysulfone

## Abstract

**Background:**

Kidney replacement therapy (KRT) is needed by patients with kidney failure to eliminate metabolic waste. When kidney transplantation is not feasible, hemodialysis (HD) is performed. Hemodiafiltration (HDF) provides a better solute clearance than conventional HD due to its higher convective dose. The clearance performances of HDF also depend on the type of dialysis membrane used. Polysulfone (PS) membranes have been used since 1965 due to their excellent resistance and biocompatibility. However, the newer generation of polymethylmethacrylate (PMMA) membranes offers comparable convective capacity with reduced albumin loss.

**Objective:**

The forthcoming study aims to compare the solute clearance of postdilution HDF using PMMA versus PS dialyzer membranes in patients with kidney failure requiring long-term HD.

**Methods:**

The literature search will use databases including PubMed, Scopus, Web of Science, Embase, and Cochrane. Two independent reviewers will select studies and assess their quality. Pooled estimates of relevant factors will be computed via a random-effects model using Stata/BE 18 (StataCorp) software. The reporting of findings will adhere to the PRISMA (Preferred Reporting Items for Systematic Reviews and Meta-Analyses) guidelines.

**Results:**

The search and screening for the systematic literature review are expected to be completed in May 2026. Data extraction, quality appraisal, and subsequent data synthesis will begin in June 2026. The review should be completed by October 2026, and the study results will be published in 2027.

**Conclusions:**

Dialyzer membrane clearance determines HD quality. Nevertheless, many middle uremic molecules are poorly cleared with conventional HD. Consequently, HDF was invented to improve their clearance. Among the most widely used dialyzer membranes, PS and PMMA are used in the newer generation of HDF. Nevertheless, no available systematic reviews have compared the efficacy of the 2 dialyzer membrane materials. Although no ideal dialyzer can efficiently remove all types of uremic toxins while retaining essential elements, improving solute clearance should benefit patients. This information could help clinicians determine which membrane to use in HDF.

**Trial Registration:**

PROSPERO CRD42024603277; https://www.crd.york.ac.uk/PROSPERO/view/CRD42024603277

**International Registered Report Identifier (IRRID):**

PRR1-10.2196/71943

## Introduction

In patients with chronic kidney disease (CKD), signs and symptoms typically develop gradually [1-3]. As kidney function declines progressively, the condition may eventually lead to kidney failure. At this stage, the kidneys can no longer effectively eliminate metabolic waste, resulting in the accumulation of toxins and disturbances in electrolyte balance [4]. The definitive treatment for kidney failure is kidney replacement therapy (KRT) through a kidney transplant. Still, this option is not always feasible or successful, necessitating other KRT modalities, namely hemodialysis (HD) or peritoneal dialysis [5-9]. HD can be divided into conventional HD, hemodiafiltration (HDF), and expanded HD [10].

Conventional HD uses solute diffusion and limited ultrafiltration, using a high-flux membrane inserted in a hollow-fiber dialyzer. Conventional HD is linked to “residual risk” resulting from the accumulation of inadequately removed medium-sized compounds. Meanwhile, HDF uses convection and diffusion to eliminate small- and medium-sized molecules. A significant distinction between HDF and conventional HD is the total ultrafiltered volume per session (dialytic convective dose). HDF can achieve a significantly higher convective dose (10- to 15-fold), leading to a 2-fold increase in middle-molecular-weight solute clearance [11,12].

HDF was initially introduced in the United States by Lee Henderson in 1973 [13]. On-line generation of substitution fluid for HDF was then implemented in the mid-1980s [14]. HDF has since undergone substantial development. See et al (2019) [15] found that HDF was associated with a significantly lower risk of all-cause mortality (adjusted hazard ratio [HR] of 0.79 and 0.88 for patients in Australia and New Zealand, respectively). An association was also found between HDF and reduced cardiovascular mortality, with an adjusted HR of 0.78 in Australian patients. Using the French Renal Epidemiology and Information Network Registry, Mercadal et al (2016) [16] found that the HRs for all-cause and cardiovascular mortality in patients treated exclusively with HDF were 0.77 and 0.66, respectively.

HDF can be conducted with dialysis membranes comprising different raw materials, including cellulose, polysulfone (PS), polyamide, polymethylmethacrylate (PMMA), and polycarbonate [17]. Clinicians must consider several factors to determine which dialyzer membrane is most suitable for dialysis: performance, biocompatibility, cytotoxicity, sterilization, and endotoxin retention [18]. This systematic review and meta-analysis will focus on the efficacy of PMMA dialyzers compared to that of PS in postdilution HDF in patients with kidney failure requiring long-term HD. A PS membrane has an asymmetric structure where the pore size enlarges from inside to outside and a thin skin layer with very small pore sizes to control solute separations. Conversely, a PMMA membrane has a uniform pore size structure, and the entire membrane controls solute separations. Therefore, the separation characteristics of PMMA are broader than those of PS membranes. Furthermore, PS membranes remove uremic toxins from the blood via diffusion and filtration, while PMMA membranes also use adsorption in addition to diffusion and filtration [19-21]. However, PMMA membranes have lower permeability than PS membranes, increasing the risk of fouling and platelet-induced membrane coagulation [22].

A dialyzer’s efficacy is assessed based on the clearance of solutes, including urea, creatinine, phosphate, uric acid, albumin, α1-microglobulin, ß2-microglobulin, myoglobin, prolactin, and κ and λ free light chains [23,24]. Our review aims to answer the following question: in patients with kidney failure requiring long-term HD, does the PMMA dialyzer membrane demonstrate a higher efficacy than the PS dialyzer membrane? The PICO (Population, Intervention, Comparison, and Outcomes) framework will be used during the article search; “P (population)” refers to patients with kidney failure requiring long-term HD, “I (intervention)” refers to HDF using PMMA dialyzer membrane, “C (comparison)” refers to HDF using the PS dialyzer membrane, and “O (outcome)” refers to the efficacy of solute clearance.

## Methods

### Overview

This review will be conducted and reported following the PRISMA-P (Preferred Reporting Items for Systematic Reviews and Meta-Analyses Protocols) guidelines (Multimedia Appendix 1) [25,26]. We will also integrate this review with the Meta-analysis of Observational Studies in Epidemiology guidelines, since the outcome will be a meta-analysis of selected observational studies [27].

### Eligibility Criteria

We will include all original articles in English based on the following criteria:

1. Studies involving adult patients diagnosed with kidney failure and requiring long-term HD.
2. Studies investigating postdilution HDF using PMMA and/or PS as dialyzer membranes.
3. Studies reporting dialysis efficacy as the primary or secondary outcome.
4. Peer-reviewed original articles, including randomized controlled trials (RCTs) and observational studies (cohort, case-control, and cross-sectional studies).

The exclusion criteria are as follows:

1. Studies involving patients with acute kidney injury, from which long-term dialysis patients’ group data cannot be extracted.
2. Studies using dialyzer membranes other than PMMA or PS and using mixed or combined membrane types, from which PMMA or PS group data cannot be extracted.
3. Studies investigating dialysis modalities other than HDF (eg, high-flux and low-flux HD, expanded HDF).
4. Any review, case study, commentary, qualitative study, or editorial.

### Outcome Measures

The review outcomes are efficacy based on solute clearance of urea, creatinine, phosphate, uric acid, α1‑microglobulin, β2-microglobulin, myoglobin, albumin, and prolactin. The efficacy will be assessed based on the solutes’ reduction ratio (RR) measured before and after the HDF session. Quantitative data will be reported as mean (SD) values.

### Information Sources

#### Electronic Search

We will systematically conduct a comprehensive literature search using various literature databases, including MEDLINE (PubMed), Web of Science, Scopus, Embase, and the Cochrane Central Register of Controlled Trials (CENTRAL) to identify eligible studies. Secondary searches will be conducted on Google Scholar and other websites, and the reference section of the included studies will also be hand-searched for additional relevant studies. Studies will be restricted to the English language. The search will be performed from selected electronic databases up to October 2025.

#### Search Strategy

The proposed search term for the first theme will be “long-term hemodialysis.” The second theme is “polymethylmethacrylate” and “polysulfone.” The third theme is “efficacy.” The exploded versions of the MeSH (Medical Subject Headings) browser for each theme will be included. All 3 search themes will be combined using the Boolean operators “OR” and “AND.” Multimedia Appendix 2 presents the detailed search terms for each database.

#### Study Selection

We will identify and remove duplicates and collate multiple reports. Two review authors (AKK and NN) will independently screen all the titles and abstracts to examine the potential studies for inclusion and exclude evidently irrelevant studies. We will identify studies from databases and remove duplicate records. We will then perform an initial screening based on the title and abstract of the articles to identify those that are irrelevant or meet the inclusion criteria. Articles deemed irrelevant based on the title and abstract will be excluded from further consideration. For the included study, we will retrieve the full-text study reports and publications, and the review authors will independently screen the full text to identify studies for inclusion and exclusion to determine their eligibility. The reasons for excluding articles, such as having an in vitro design and having different interventions, HD modalities, patient groupings, outcomes, or measurements, will be documented. Articles that meet all inclusion criteria and are not excluded will be included in the review.

We will resolve any disagreement through discussion. If no consensus is reached, the other 2 authors will act as arbiters. We will record the selection process in sufficient detail to complete a PRISMA (Preferred Reporting Items for Systematic Reviews and Meta-Analyses) flow diagram and construct a table describing the characteristics of the excluded studies [25,26]. Rayyan (Qatar Computing Research Institute) will be used to store, organize, and manage all the articles identified from databases.

#### Data Extraction and Management

We will use a standardized data extraction form created by the Microsoft Excel Spreadsheet software for study characteristics and outcome data. Two review authors (CGA and NN) will independently extract outcome data from the included studies. Discrepancies in determining data usability will be resolved by discussion or by involving the other 2 review authors. The usability of outcome data will be documented in the characteristics of included studies. Data will be considered unusable if any of the following apply: (1) a nonstandard formula or procedure is used to calculate the RR; (2) RR is reported as median (IQR), and conversion to mean (SD) is not feasible; (3) results are presented only in graphical form without sufficient resolution or accompanying numerical data; (4) inconsistent or incomplete reporting of outcome data across comparison groups; (5) the HDF procedure is not adequately described, particularly concerning the dialysis modality (eg, postdilution vs predilution), membrane type, and dialysis settings; (6) the type of membrane used is not reported or is ambiguous; and (7) key demographic or baseline characteristics of participants (eg, age, sex, and dialysis duration) are not reported. Studies meeting the eligibility criteria but excluded due to unusable data will be listed in the final review along with specific reasons for exclusion, to ensure transparency and minimize selective reporting bias.

We will recheck that data are entered correctly by comparing the systematic review data with the study reports. The following study characteristics from the included studies will be extracted:

- Title, first author, study country based on the study setting, region, and publication year.
- Methods: study design, data source, total study duration, and analysis method.
- Participants: number of patients, mean and/or median age, age range, and sex.
- Outcome: efficacy based on solute clearance, which is the measurement of RR of each solute such as urea, creatinine, phosphate, uric acid, α1-microglobulin, β2-microglobulin, myoglobin, albumin, and prolactin, which were measured before and after HDF.
- Exposures or risks: CKD etiology, dialysis vintage, and comorbidity. However, whenever data are applicable, we will extract the data on other exposure risks or modifiable factors, control conditions, and adjustment variables.

If there is any unclear or missing data for extraction, we will contact the corresponding author of the original studies to request clarification or check whether additional data can be made available upon request. If there is no response from the corresponding author within a reasonable time frame, the study will be excluded from the analysis, with the reason for exclusion documented in the review. We will also assess the potential impact of missing data on the overall findings through sensitivity analysis, where feasible. We will also consider the risk of bias due to incomplete data in the overall quality assessment.

#### Quality Assessment

Two review authors will independently assess the risk of bias depending on the study design (AKK and AW). For RCTs, we will use the Cochrane Risk of Bias 2 (RoB 2) tool, which evaluates five domains: (1) bias due to the randomization process, (2) deviation from intended intervention, (3) missing outcome data, (4) measurement of outcomes, and (5) selection of the reported result. Each domain will be rated as “low risk,” “some concerns,” or “high risk,” and an overall risk of bias judgment will be assigned [28].

For nonrandomized intervention studies, we will use the Risk Of Bias In Non-randomized Studies of Interventions (ROBINS-I) tool. The risk of bias will be assessed across several domains: (1) bias due to confounding, (2) bias in participant selection, (3) bias in classification of the intervention, (4) bias from deviations in the intended interventions, (5) bias due to missing outcome data, (6) bias in outcome measurement, and (7) bias in the selection of reported results. The risk of bias for each domain will be rated as low, moderate, serious, or critical. An overall risk of bias judgment will also be made [29]. In the sensitivity analysis, we will exclude trials assessed as high risk of bias with the RoB 2 tool and those rated as serious or critical risk of bias with the ROBINS-I tool. This will allow us to assess whether the inclusion of these studies significantly influences the pooled effect estimates.

### Statistical Analysis

#### Data Analysis and Statistical Analysis

The primary outcome of our study is the efficacy, which will be based on solute clearance, based on the RR of each solute. RR will be determined using laboratory parameters, which include serum concentrations of urea, creatinine, phosphate, uric acid, α1-microglobulin, β2-microglobulin, myoglobin, albumin, and prolactin, measured before and after HDF. Quantitative data will be reported as mean (SD) values. Since RRs are measured using the same scale across all included studies, we will use the mean difference as the effect measure. For parametric comparisons, paired Student *t* tests followed by Bonferroni post hoc tests will be used. A *P* value of <.05 will be considered statistically significant.

Although both RCTs and nonrandomized studies will be included in this systematic review, a meta-analysis will be conducted separately for each study design to account for their inherent methodological differences. Meta-analyses will be conducted using a random-effects model to account for potential variability and heterogeneity among studies. Given the inclusion of nonrandomized studies, we acknowledge the potential for confounding and selection bias. To address this, we will prioritize adjusted effect estimates (eg, those derived from multivariable regression models or propensity score analyses) where available. Additionally, we will conduct subgroup or sensitivity analyses by excluding studies judged to be at overall high risk of bias. If 10 or more studies are included, we will consider performing meta-regression to explore potential effect modifiers. A narrative synthesis will be provided for outcomes not amenable to quantitative synthesis.

#### Heterogeneity Assessment

Assessing heterogeneity is critical in a meta-analysis, as it enables the evaluation of the degree of inconsistency or variability among the results of individual studies. This review will evaluate heterogeneity using the *I*^2^ and Q statistics. The *I*^2^ statistic will be used to quantify the impact of heterogeneity, with percentages of around 25%, 50%, and 75% representing a low, moderate, and high degree of heterogeneity, respectively [30]. The statistical Q test determines whether significant heterogeneity exists among the studies. The significance level for the Q test is set at 0.01 in this review [31]. If significant heterogeneity is detected using the Q test or *I*^2^ index (>50%), we will explore potential heterogeneity sources using subgroup analyses and meta-regression. We will also explore possible causes (for example, differences in study quality, participants, or outcome assessments) and evaluate the studies based on their methodological characteristics to determine whether differences in these characteristics can explain the degree of heterogeneity and whether a meta-analysis is appropriate.

#### Assessment of Publication Bias

We will create and examine a funnel plot to explore possible small study biases if we can pool over 10 studies in 1 meta-analysis. The number of studies missing from the funnel plot will be estimated. The effect size after inputting these missing studies will be estimated using the trim-and-fill method. This simple estimation approach proposed by Duval and Tweedie [32] involves trimming off the asymmetric outlying part of the funnel, using the symmetric remainder to estimate the true funnel center, and replacing the trimmed studies and their counterparts around the center. Other methods to assess the publication bias will include the Begg rank correlation and the Egger weighted regression method test [33,34].

### Ethical Considerations

We registered this systematic review in PROSPERO (systematic review registration: CRD42024603277). Since this review will use published data, the need for ethical approval is waived in accordance with local guidelines. The systematic review will focus on determining whether the PMMA or the PS dialyzer membrane has a higher efficacy in patients with kidney failure requiring long-term HD.

## Results

The search and screening for the systematic literature review is expected to conclude in May 2026. Data extraction, quality appraisal, and subsequent data synthesis will begin in June 2026. The review should be completed by October 2026, and the study results will be published in 2027.

## Discussion

PS is an amorphous polymer with excellent thermal and chemical stability [35]. The PS membrane is asymmetric, implying that for a membrane thickness of 30 microns, a 1-micron layer controls the separation process, while the remainder has structural functions. Conversely, the PMMA membrane is symmetric, with the whole thickness involved in the separation process [21]. The PMMA membrane adds adsorption to the existing HD diffusion and convection processes. While diffusion and convection remove small and medium-sized molecules, adsorption enables the removal of medium- and high-molecular-weight molecules [22].

The removal of toxins and waste products from the patient’s blood defines the adequacy of HD. Adequate dialysis can improve patient survival and quality of life [36-39]. Kt/V calculation is the most widely used method to assess dialysis adequacy, where K is the effective dialyzer solute clearance, t is the duration of the dialysis session, and V is the solute distribution volume [40]. Currently, small solute clearance is regarded as the best measure of HD adequacy. Kt/V urea, also known as fractional urea clearance, is a widely used tool to assess dialysis efficacy in daily clinical practice [41].

The use of urea as the sole representation of dialysis adequacy tends to oversimplify this concept. In this context, urea does not represent the kinetic behavior of other solutes, including inorganic solutes such as phosphate, middle molecules such as β2-microglobulin, and protein-bound uremic toxins such as p-cresyl sulfate, whose detrimental effects are linked with inflammation, oxidative stress, and cardiovascular disease [42-47]. Hence, the concept of adequacy is evolving to include nonurea parameters [48].

HD adequacy is partly determined by dialyzer membrane clearance [49]. Moreover, membrane properties, including pore size, wall thickness, and surface area, determine solute clearance. The uremic toxins HD removes are divided into four categories: (1) small water-soluble compounds (eg, urea) with a size of <500 Da, (2) a middle-molecular-weight of 500-15,000 Da, (3) large molecules with a size of >15,000 Da, and (4) protein-bound molecules (eg, p-cresol of 108 Da). However, when describing the in vitro performance of dialyzers, many studies only considered the results obtained with small solutes and middle-molecular-weight molecules [50].

Middle-molecular-weight molecules include several cytokines, adipokines, growth factors, and other signaling proteins significantly elevated in dialysis patients compared to that in people with normal kidney function. The serum levels of interleukin (IL)-1β, IL-6, and IL-18, and κ and λ free light chains are also elevated in patients with advanced CKD [51]. These molecules play key roles in the inflammation-cardiovascular disease pathway and are linked with chronic inflammation, atherosclerosis, structural cardiac disease, and protein-energy wasting [52,53].

While conventional HD can effectively remove small uremic molecules (<0.5 kDa), including urea, it poorly removes middle-molecular-weight molecules in the 0.5-60 kDa range. Thus, HDF was invented to improve the clearance of middle-molecular-weight molecules [54]. The clinical application of on-line generation of replacement fluid HDF has been limited in the United States and has predominantly advanced in Europe. Increasing numbers of patients in Japan and other Asian countries have recently opted for HDF treatment [55,56].

The HDF removal efficiency is mainly determined by the replacement fluid volume, while the removal characteristic is affected by the dilution method. Postdilution HDF, when combined with a protein-nonleakage hemodiafilter, is superior in removing small to middle-molecular-weight molecules. Conversely, predilution HDF with a protein-leakage–type hemodiafilter is preferred for removing middle- to high-molecular weight molecules [57]. Predilution HDF efficiently removes middle-molecular-weight molecules of up to α1-microglobulin (26-30 kDa). However, the biological activity of α1-microglobulin is unclear, and the uremic toxins in its vicinity are also unknown. Meanwhile, postdilution HDF increases the removal efficiency for molecular weights up to those of β2-microglobulin (11-12 kDa), which has been established as a uremic toxin [57].

Views differ on the impact of dialyzer characteristics on patient outcomes and the characteristics that the clinician should prioritize. The impact also relies on the dialyzer’s application, where convective therapies such as HDF outperform diffusive therapies such as HD [58]. Although no ideal dialyzer can efficiently remove all types of uremic toxins while retaining vitamins and other essential elements, expanding the range of uremic toxin clearance should benefit patients. Nevertheless, further research is required to demonstrate the superiority of these new dialyzers [59].

Our study will include stable patients with kidney failure undergoing thrice weekly HD sessions. We recognize that the duration of each dialysis session (in minutes) might differ between studies. Therefore, we will only include studies with similar dialysis prescriptions and the same modality, postdilution HDF, to limit bias. To our knowledge, this study will be the first to conduct a systematic review and meta-analysis of the available evidence regarding comparing clearance between PMMA and PS dialyzer membranes for postdilution HDF in patients with kidney failure.

Our study may have several limitations. Even if the studies used the same membrane material (PS or PMMA), if they used dialyzers from different manufacturers or the same manufacturer but different versions (type, design, or specification), bias could result from different clearance capacities. Second, clearance is subject to methodological heterogeneity, as the time point for each blood sampling collection might differ between studies. We will not specify time points as inclusion criteria due to the risk of eliminating potentially relevant studies.

Third, our study will not assess some aspects potentially affecting the fluid removal rate. We will not evaluate the patients’ primary kidney disease or other health problems potentially affecting the blood protein level (albumin). Moreover, we will not assess the quality of blood vessels or HD lumen catheters used for dialysis, which potentially affect dialysis efficiency through resistance and subsequently influence the fluid removal rate.

In summary, our systematic review and meta-analysis may help compare the solute clearance of PMMA and PS dialyzer membranes for postdilution HDF in people with kidney failure. The results may help clinicians determine the most suitable membrane for HDF for their routine HD practice. The data may also provide a starting point for further research on the impacts of various dialyzer membranes on the HDF solute removal rate.

## Appendix

Multimedia Appendix 1 PRISMA-P checklist.

Multimedia Appendix 2 Search terms.

## Abbreviations

CKD: chronic kidney disease
HD: hemodialysis
HDF: hemodiafiltration
HR: hazard ratio
IL: interleukin
KRT: kidney replacement therapy
MeSH: Medical Subject Headings
PICO: Population, Intervention, Comparison, and Outcomes
PMMA: polymethylmethacrylate
PRISMA: Preferred Reporting Items for Systematic Reviews and Meta-Analyses
PRISMA-P: Preferred Reporting Items for Systematic Reviews and Meta-Analyses Protocols
PS: polysulfone
RCT: randomized controlled trial
RoB 2: Cochrane Risk of Bias 2
ROBINS-I: Risk Of Bias In Non-randomized Studies of Interventions
RR: reduction ratio
