# Supplementary material for: Comparing Clearances of Polymethylmethacrylate and Polysulfone Dialyzer Membranes for Postdilution Hemodiafiltration in Patients With Kidney Failure: Protocol for a Systematic Review and Meta-Analysis
Source: JMIR Res Protoc. 2025 Jun 26;14:e71943. doi: 10.2196/71943 (PMC12246762; doi:10.2196/71943)
Supplement: Multimedia Appendix 2 [file resprot_v14i1e71943_app2.docx]

Table S1. Proposed Search Terms

| **Databases** | **Search Terms** |
| --- | --- |
| MedLine | ("hemodialysis"[Title/Abstract] OR “haemodialysis”[Title/Abstract]) OR "dialysis"[Title/Abstract] OR ("dialyzer"[Title/Abstract] OR "dialyser"[Title/Abstract]) OR ("hemodiafiltration"[Title/Abstract] OR “haemodiafiltration”[Title/Abstract]) OR ("hemodiafilter"[Title/Abstract] OR “haemodiafilter”[Title/Abstract]) OR ("hemofilter"[Title/Abstract] OR “haemofilter”[Title/Abstract])  AND  ("polymethylmethacrylate"[Title/Abstract] OR "polymethyl methacrylate"[Title/Abstract] OR "PMMA"[Title/Abstract]) AND ("polysulfone"[Title/Abstract])  AND  ("efficacy"[Title/Abstract] OR "efficiency"[Title/Abstract] OR "reduction rate"[Title/Abstract] OR "reduction ratio"[Title/Abstract] OR “clearance”[Title/Abstract] OR “removal”[Title/Abstract] OR "urea"[Title/Abstract] OR "creatinine"[Title/Abstract] OR "blood urea nitrogen"[Title/Abstract] OR "albumin"[Title/Abstract] OR "microglobulin"[Title/Abstract] OR "clinical evaluation"[Title/Abstract] OR ("dialyzer performance"[Title/Abstract] OR "dialyser performance"[Title/Abstract])) |
| Scopus | TITLE-ABS-KEY("h*modialysis" or "dialysis" or "dialy?er" or "hemodiafil*" or "h*mofilter")  AND  TITLE-ABS-KEY("polymethylmethacrylate" OR "polymethyl methacrylate" OR "PMMA") AND "polysulfone"  AND  TITLE-ABS-KEY("efficacy" OR "efficiency" OR "reduction rate" OR "reduction ratio" OR “clearance” OR “removal” OR "urea" OR "creatinine" OR "blood urea nitrogen" OR "albumin" OR "microglobulin" OR "clinical evaluation" OR "dialy?er performance")  AND ( LIMIT-TO ( DOCTYPE , "ar" ) ) |
| CENTRAL* | "hemodialysis"[Title/Abstract/Keyword] OR "haemodialysis"[Title/Abstract/Keyword]) OR "dialysis"[Title/Abstract/Keyword] OR "dialyzer"[Title/Abstract/Keyword] OR "dialyser"[Title/Abstract/Keyword]) OR "hemodiafilter"[Title/Abstract/Keyword] OR "haemodiafilter"[Title/Abstract/Keyword] OR "hemodiafiltration"[Title/Abstract/Keyword] OR "haemodiafiltration"[Title/Abstract/Keyword] "hemofilter"[Title/Abstract/Keyword] OR "haemofilter"[Title/Abstract/Keyword]  AND  ("polymethylmethacrylate"[Title/Abstract/Keyword] OR “polymethyl methacrylate” [Title/Abstract/Keyword] OR "PMMA"[Title/Abstract/Keyword]) AND (“polysulfone”) [Title/Abstract/Keyword]  AND  ("efficacy"[Title/Abstract/Keyword] OR "efficiency"[Title/Abstract/Keyword] OR "reduction rate"[Title/Abstract/Keyword] OR "reduction ratio"[Title/Abstract/Keyword] OR “clearance” [Title/Abstract/Keyword] OR “removal” [Title/Abstract/Keyword] OR "urea"[Title/Abstract/Keyword] OR "creatinine"[Title/Abstract/Keyword] OR "blood urea nitrogen"[Title/Abstract/Keyword] OR "albumin"[Title/Abstract/Keyword] OR "microglobulin"[Title/Abstract/Keyword] OR "clinical evaluation"[Title/Abstract/Keyword] OR ("dialyzer performance"[Title/Abstract/Keyword] OR "dialyser performance"[Title/Abstract/Keyword])) |
| Web of Science | AB=("hemodialysis" OR "haemodialysis" OR "dialysis" OR "dialyzer" OR "dialyser" OR "hemodiafil*" OR "haemodiafil*" OR "hemofilter" OR "haemofilter")    AND  AB=("polymethylmethacrylate" OR "polymethyl methacrylate" OR "PMMA" AND "polysulfone")  AND  AB=(("efficacy" OR "efficiency" OR "reduction rate" OR "reduction ratio" OR "clearance" OR "removal" OR "urea" OR "creatinine" OR "blood urea nitrogen" OR "albumin" OR "microglobulin" OR "clinical evaluation") OR ("dialyzer performance" OR "dialyser performance")) |
| EMBASE | (("hemodialysis" or "haemodialysis" or "dialysis" or "dialyser" or "hemodiafil*" or "haemodiafil" or "hemofilter" or "haemofilter") and ("polymethylmethacrylate" or "polymethyl methacrylate" or "PMMA") and "polysulfone" and ("efficacy" or "efficiency" or "reduction rate" or "reduction ratio" or "clearance" or "removal" or "urea" or "creatinine" or "blood urea nitrogen" or "albumin" or "microglobulin" or "clinical evaluation" or ("dialyzer performance" or "dialyser performance"))).ti,ab. |

*CENTRAL: Cochrane Central Register of Controlled Trials
